# Multi-ancestry genome- and phenome-wide association studies of diverticular disease in electronic health records with natural language processing enriched phenotyping algorithm

**DOI:** 10.1371/journal.pone.0283553

**Published:** 2023-05-17

**Authors:** Yoonjung Yoonie Joo, Jennifer A. Pacheco, William K. Thompson, Laura J. Rasmussen-Torvik, Luke V. Rasmussen, Frederick T. J. Lin, Mariza de Andrade, Kenneth M. Borthwick, Erwin Bottinger, Andrew Cagan, David S. Carrell, Joshua C. Denny, Stephen B. Ellis, Omri Gottesman, James G. Linneman, Jyotishman Pathak, Peggy L. Peissig, Ning Shang, Gerard Tromp, Annapoorani Veerappan, Maureen E. Smith, Rex L. Chisholm, Andrew J. Gawron, M. Geoffrey Hayes, Abel N. Kho

**Affiliations:** 1 Department of Medicine, Northwestern University Feinberg School of Medicine, Chicago, IL, United States of America; 2 Center for Genetic Medicine, Northwestern University Feinberg School of Medicine, Chicago, IL, United States of America; 3 Center for Health Information Partnerships, Northwestern University Feinberg School of Medicine, Chicago, IL, United States of America; 4 Department of Preventive Medicine, Northwestern University Feinberg School of Medicine, Chicago, IL, United States of America; 5 College of Medicine, Mayo Clinic, Rochester, MN, United States of America; 6 Geisinger, Danville, PA, United States of America; 7 Icahn School of Medicine at Mount Sinai, New York, NY, United States of America; 8 Partners Healthcare, Charlestown, MA, United States of America; 9 Kaiser Permanente Washington Health Research Institute, Seattle, Washington, United States of America; 10 Departments of Biomedical Informatics and Medicine, Vanderbilt University, Nashville, TN, United States of America; 11 The Charles Bronfman Institute for Personalized Medicine, Icahn School of Medicine at Mount Sinai, New York, NY, United States of America; 12 Office of Research Computing and Analytics, Marshfield Clinic Research Institute, Marshfield, WI, United States of America; 13 Department of Healthcare Policy and Research, Weill Cornell Medical College, New York, NY, United States of America; 14 Center for Precision Medicine Research, Marshfield Clinic Research Institute, Marshfield, WI, United States of America; 15 Department of Biomedical Informatics, Columbia University, New York, NY, United States of America; 16 Division of Molecular Biology and Human Genetics, Department of Biomedical Sciences, Faculty of Medicine and Health Sciences, Stellenbosch University, Stellenbosch, South Africa; 17 Department of Medicine, Gastroenterology, Duke University, Durham, NC, United States of America; 18 Division of Gastroenterology, Hepatology & Nutrition, University of Utah, Salt Lake City, UT, United States of America; 19 Department of Anthropology, Northwestern University, Evanston, IL, United States of America; 20 Division of General Internal Medicine and Geriatrics, Department of Medicine, Northwestern University Feinberg School of Medicine, Chicago, IL, United States of America; Brigham and Women’s Hospital and Harvard Medical School, UNITED STATES

## Abstract

**Objective:**

Diverticular disease (DD) is one of the most prevalent conditions encountered by gastroenterologists, affecting ~50% of Americans before the age of 60. Our aim was to identify genetic risk variants and clinical phenotypes associated with DD, leveraging multiple electronic health record (EHR) data sources of 91,166 multi-ancestry participants with a Natural Language Processing (NLP) technique.

**Materials and methods:**

We developed a NLP-enriched phenotyping algorithm that incorporated colonoscopy or abdominal imaging reports to identify patients with diverticulosis and diverticulitis from multicenter EHRs. We performed genome-wide association studies (GWAS) of DD in European, African and multi-ancestry participants, followed by phenome-wide association studies (PheWAS) of the risk variants to identify their potential comorbid/pleiotropic effects in clinical phenotypes.

**Results:**

Our developed algorithm showed a significant improvement in patient classification performance for DD analysis (algorithm PPVs ≥ 0.94), with up to a 3.5 fold increase in terms of the number of identified patients than the traditional method. Ancestry-stratified analyses of diverticulosis and diverticulitis of the identified subjects replicated the well-established associations between *ARHGAP15* loci with DD, showing overall intensified GWAS signals in diverticulitis patients compared to diverticulosis patients. Our PheWAS analyses identified significant associations between the DD GWAS variants and circulatory system, genitourinary, and neoplastic EHR phenotypes.

**Discussion:**

As the first multi-ancestry GWAS-PheWAS study, we showcased that heterogenous EHR data can be mapped through an integrative analytical pipeline and reveal significant genotype-phenotype associations with clinical interpretation.

**Conclusion:**

A systematic framework to process unstructured EHR data with NLP could advance a deep and scalable phenotyping for better patient identification and facilitate etiological investigation of a disease with multilayered data.

## Introduction

Diverticular disease (DD) is the most common morphological defect of the intestinal tract and the fifth most important gastrointestinal (GI) disorder in terms of medical cost as high as >$5.4 billion in the United States [1–3]. DD usually indicates asymptomatic diverticulosis (the mere presence of diverticula, a pouch-like protrusion in the colonic wall), but also includes diverticulitis (acute or chronic inflammation of diverticula) and its clinical complications [4]. Diverticulitis occurs in approximately 4% to 15% of patients with diverticula and has a high reoccurrence rate, which is associated with fever, abdominal pain, leukocytosis, and potentially life-threatening peritonitis [4–7].

DD has long been regarded as a disease of Western countries [8]; North America has the highest prevalence of DD, affecting approximately one-third of the population older than 45, and up to 67% over 65 [6, 9]. However, in recent decades, virtually all countries worldwide are observing an increasing burden of DD irrespective of their economic developmental or demographical variability [10–13]. Dietary intake of low fiber, processed foods, and red meats has been implicated as potential causes of DD [8, 14], but this idea is still controversial [15, 16].

As with most medical conditions, current evidence supports a complex interplay of both environmental and genetic factors in the pathophysiology of DD. Twin studies reveal that the genetic heritability of DD is estimated to be up to 53% (95% CI, 45–61%) [5]. To date, three GWAS have identified 52 genetic susceptibility loci associated with DD [17–19].

A significant challenge to its etiologic investigation is that approximately 75% to 90% of diverticulosis patients remain asymptomatic until presenting with diverticulitis [20], making it difficult to self-identify or detect the disorder in clinical setting. In acute cases, a computed tomography (CT) imaging of the abdomen is most often used in the evaluation of diverticulitis, but it may not be completely diagnostic in cases of early or mild diverticulitis [21]. Currently, the definitive ascertainment of the presence or absence of DD depends on colonoscopy results [21–23], but this requirement suffers from incomplete patient compliance given current screening guidelines [24].

To address these challenges, we developed an automated phenotyping algorithm that incorporated natural language processing (NLP) technique to efficiently identify the presence or absence of diverticulosis or diverticulitis utilizing both structured and unstructured data from the electronic health records (EHR). By integrating heterogeneous EHR data sources, we aim to present a scalable framework to perform EHR-powered GWAS and phenome-wide association studies (PheWAS) to systematically investigate the genetic epidemiology of DD.

## Methods

### NLP-enriched phenotyping algorithm for DD

Genome-wide genotype data of 38,827 individuals from 9 EHR-linked biobanks and phenotype data including their demographic, clinical diagnosis, colonoscopy reports of 99,185 individuals were collected from 12 EHR-linked biobanks from the electronic Medical Records and Genomics (eMERGE) network [25, 26]. The details of genotyping, imputation, and quality control processes are available in **S1 File**.

**Fig 1:**
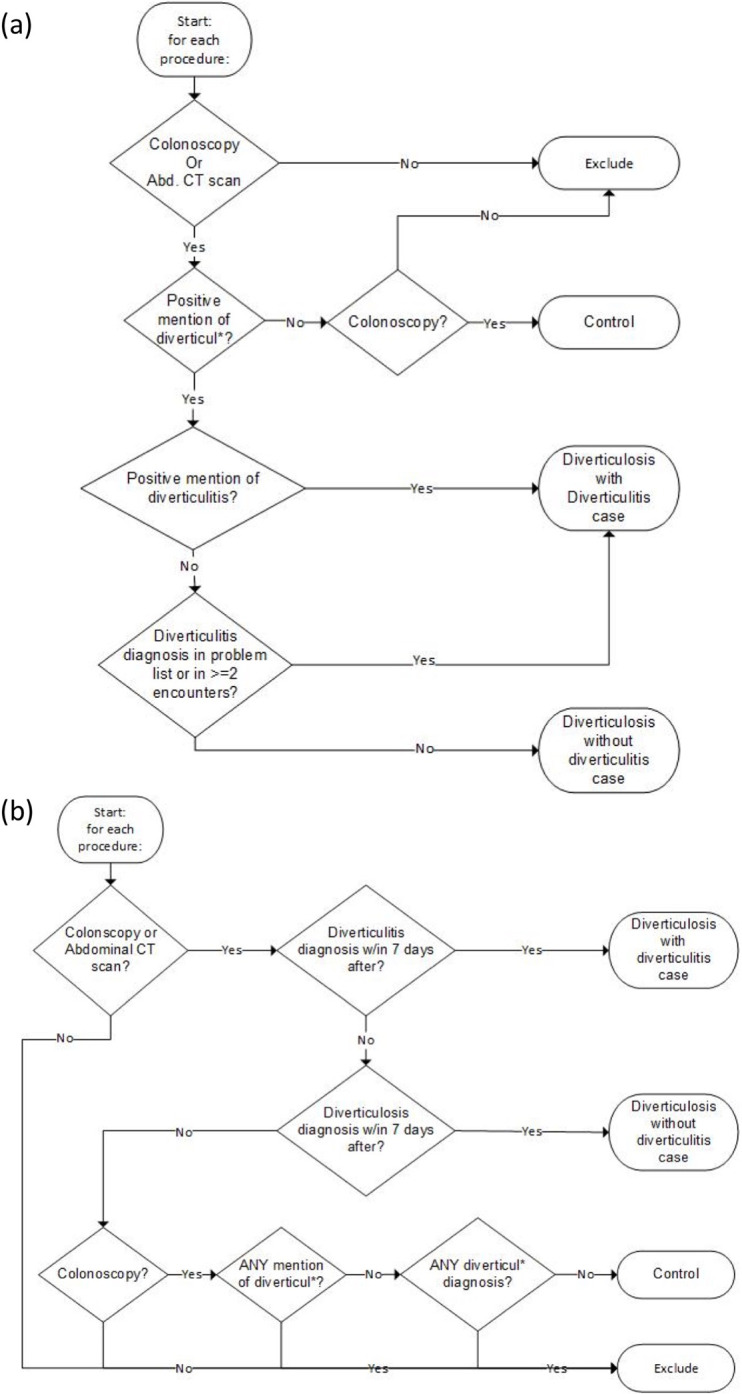
Natural language processing (NLP)-enriched phenotyping algorithms for diverticular disease (DD) cases and controls. (a) The NLP-driven phenotyping algorithm used in five medical institutions in the eMERGE network (NU, VU, Geisinger, KPWA/UW, Mayo clinic). (b) The structured data-driven phenotyping algorithm was used in two eMERGE sites (Marshfield, Mount Sinai).

We developed two different phenotyping algorithms while accounting for data availability at each implementing site. For patients with physician reports in the EHR, the first NLP-driven algorithm scanned the unstructured text of colonoscopy or abdominal imaging reports to identify DD. The algorithm considered any subject that had any positively asserted mention of “diverticul*” in those reports to have diverticulosis, and a positively asserted mention of “diverticulitis” was considered to have diverticulosis with diverticulitis. We used the ConText algorithm [27], an updated NegEx tool, to detect negated mentions of either diverticulosis or diverticulitis and supplemented the results with diagnostic and procedure codes additionally **(Fig 1A)**.

For the sites where only a limited subset of these imaging reports were available, the algorithm alternatively used International Classification of Disease 9^th^ revision (ICD-9) codes that started with 562(‘Diverticulosis and diverticulitis’ category), assigned within 7 days after a colonoscopy or abdominal imaging, to select diverticulosis cases. The result was supplemented with NLP components when physician reports were available **(Fig 1B)**. Additional criteria to define ‘diverticulosis’ and ‘diverticulitis’ are detailed in **S1 File**.

We validated the algorithm performance by a standardized chart review of randomly selected patients’ charts. Trained clinicians and chart reviewers reviewed a total of 364 individuals’ records to assess the positive predictive value (PPV) of our developed algorithms, using established guidelines [28] from four data collection sites.

### Genome-wide association tests

Multi-ancestral (MA) GWAS was conducted on the identified subjects from the 9 sites that implemented our phenotyping algorithms **(Table 1)**. We used logistic regression (PLINK v.1.9 [29]), adjusting for sex, age at colonoscopy, study site, and the first 10 principal components of ancestry. To test for associations with diverticulosis, we compared the patients with diverticulosis, either with or without diverticulitis, to the healthy control patients without any evidence of diverticulosis or diverticulitis. To test for associations with diverticulitis, we excluded any diverticulosis patients without diverticulitis records, and compared the patients with diverticulitis (presenting both diverticulosis and diverticulitis) to the healthy control patients. Similar GWASs were repeated in European ancestry (EA) and African ancestry (AA) participants separately, which are the two largest ancestral groups available.

**Table 1:** Demographic characteristics of the patients by each eMERGE site. Patients with diverticulitis are a subset of the people with diverticulosis.

Site*	Subjects (N)	Diverticulosis Cases	Diverticulitis Cases	Healthy Controls**	Average Age (mean +/- SD)	Average BMI (mean +/- SD)	Sex (Female)	Race (EA)***	Race (AA)***
**All**	21777	12577(57.8%)	1265(10.1%)	9200(42.2%)	62.5(+/-12.2)	29.4(+/-6.8)	11891(54.6%)	19211(88.2%)	2322(10.7%)
**Columbia University**	523	39(7.5%)	39(100.0%)	484(92.5%)	63.0(+/-15.4)	27.9(+/-10.4)	283(54.1%)	311(59.5%)	168(32.1%)
**Kaiser Permanente Washington/University of Washington**	862	448(52.0%)	120(26.8%)	414(48.0%)	74.4(+/-9.8)	27.3(+/-5.4)	499(57.9%)	789(91.5%)	33(3.8%)
**Geisinger**	1603	1093(68.2%)	170(15.6%)	510(31.8%)	65.6(+/-12.4)	30.7(+/-7.4)	596(37.2%)	1595(99.5%)	6(0.4%)
**Harvard**	1651	884(53.5%)	72(8.1%)	767(46.5%)	57.4(+/-12.7)	28.8(+/-6.4)	943(57.1%)	1535(93.0%)	90(5.5%)
**Marshfield**	3324	2214(66.6%)	266(12.0%)	1110(33.4%)	62.8(+/-10.0)	29.5(+/-5.7)	1971(59.3%)	3306(99.5%)	2(0.1%)
**Mayo**	5417	3275(60.5%)	251(7.7%)	2142(39.5%)	65.1(+/-10.9)	29.3(+/-6.4)	2509(46.3%)	5379(99.3%)	12(0.2%)
**Mount Sinai**	1133	416(36.7%)	121(29.1%)	717(63.3%)	59.0(+/-10.2)	30.5(+/-7.4)	694(61.3%)	339(29.9%)	773(68.2%)
**NU**	1933	993(51.4%)	77(7.8%)	940(48.6%)	57.6(+/-11.4)	28.8(+/-7.4)	1496(77.4%)	1660(85.9%)	265(13.7%)
**VU**	5331	3215(60.3%)	149(4.6%)	2116(39.7%)	60.8(+/-13.0)	29.6(+/-7.3)	2900(54.4%)	4297(80.6%)	973(18.3%)

*Sites: GHC/UW = Group Health Cooperative/University of Washington, NU = Northwestern University, VU = Vanderbilt University.

** Without diverticulosis or diverticulitis

***Race & Ethnicity categories are mutually exclusive: EA = European American, AA = Black or African American; <1% Other race.

We annotated the significant GWAS loci with eQTL, deleteriousness score (CADD score [30]), and potential regulatory functions (RegulomeDB score [31]) using the GTEx v7 database. A subsequent conditional analysis was performed within a window of ±1Mb of the genome-wide significant GWAS variants using genome-wide complex trait analysis (GCTA) v.1.26 [32].

### Evaluation of our NLP-enriched phenotyping algorithm for DD

We compared our NLP-enriched phenotyping algorithm results against the results of an ICD-based phenotyping method that has been commonly implemented in previous GWASs of DD [17–19]. Using the phecode map v1.2 [33] for DD (ICD-9 562), we compared the numbers of DD patients identified by each algorithm within our multicenter EHR data. We excluded patients with any related gastrointestinal manifestations such as ‘ulcerative enterocolitis’(ICD-9 556), ‘regional enteritis’(ICD-9 558), ‘volvulus of intestine’(ICD-9 560.2), etc. to avoid classification bias **(S1 Table)**.

### PheWAS

We conducted PheWAS of independent GWAS-significant SNPs with suggestive threshold (GWAS p-value<1E-06 and LD r^2^<0.1) grouped by ancestry [34]. We retrieved the diagnoses of the 91,166 MA participants, including ICD-9 and 10 codes, whichever available at the time of analysis. With a minimum of 30 cases per phenotype [34], logistic regression between the GWAS SNPs and each phecode was performed with the adjustment for the first 10 PCs, and participation sites, through the *PheWAS* R package [34]. A false discovery rate (FDR)< 0.05 was used for reporting significance.

We also conducted PheWAS of the 52 reported GWAS susceptibility loci from the three existing GWASs of DD [17–19]. The genomic positions of the 52 loci were converted to GrCh37/hg19 (40 loci from Maguire et al. [17], 12 loci from Schafmayer et al. [18]), including three proxy variants (R^2^ > 0.5) available in our genotype data **(S2 Table).**

## Results

### Performance of NLP-enriched phenotyping algorithm

Compared to a gold standard of manual clinical chart review, the overall PPV of our phenotyping algorithm for diverticulosis cases (with/without diverticulitis) was 0.96, and 0.94 for controls without diverticulosis or diverticulitis **(Table 2)**. We identified 21,777 study participants using the developed algorithm without missing covariate data. Of these, we identified 12,577 diverticulosis cases with or without diverticulitis, of which 1,265 were diverticulitis cases, and 9,200 controls without diverticulosis or diverticulitis in the entire MA discovery cohort **(Table 1)**.

**Table 2:** Phenotyping algorithm validation and comparison of two phenotyping algorithms for diverticular diseases by site, out of 21,777 subjects with colonoscopy reports.

	Diverticulosis Cases Identified		Diverticulitis Cases Identified		Healthy Controls Identified		Evaluation of our algorithm		
	NLP-enriched phenotyping algorithm	(Traditional ICD-based phenotyping algorithm)*	NLP-enriched phenotyping algorithm	(Traditional ICD-based phenotyping algorithm)	NLP-enriched phenotyping algorithm	(Traditional ICD-based phenotyping algorithm)	Cases reviewed	Controls Reviewed	PPV** (case/control)
**All**	12,577	(3,591)	1,265	(1,201)	9,200	(13,633)	225	139	0.96/0.94
**Columbia University**	39	(164)	39	(40)	484	(274)	NA	NA	NA
**Kaiser Permanente Washington/University of Washington**	448	(227)	120	(117)	414	(465)	NA	NA	NA
**Geisinger**	1,093	(401)	170	(170)	510	(869)	34	33	0.97/0.94
**Harvard**	884	(482)	72	(79)	767	(842)	NA	NA	NA
**Marshfield**	2,214	(655)	266	(263)	1,110	(1,964)	50	50	1.00/1.00
**Mayo**	3,275	(695)	251	(291)	2,142	(3,255)	NA	NA	NA
**Mount Sinai**	416	(231)	121	(80)	717	(654)	NA	NA	NA
**NU**	993	(86)	77	(46)	940	(1,558)	91	56	0.98/0.89
**VU**	3,215	(650)	149	(115)	2,116	(3,752)	50	NA	0.88/NA

*This is for comparison purpose. Our main analysis did not utilize the samples identified by this ICD-based phenotyping algorithm.

**PPV = positive predictive value of the phenotyping algorithms overall, and by site, where cases are patients with diverticulosis (either with or without diverticulitis) and controls are patients without diverticulosis (nor diverticulitis), identified by the phenotyping algorithms.

### Evaluation of NLP-enriched phenotyping vs. ICD-based phenotyping

We identified more cases and controls using ICD-based phenotyping, than with NLP-enriched phenotyping, due to the lower availability of report data: 3,313 diverticulitis cases and 45,111 healthy controls were found with ICD-based phenotyping. However, out of 21,777 subjects with imaging reports data, ICD-based phenotyping identified only 3,591 of them as diverticulosis cases whereas our NLP-enriched algorithm identified 12,577 diverticulosis cases. For diverticulitis, our NLP-enriched algorithm identified 1,265 patients and ICD-based phenotyping identified 1,201 patients **(Table 2)**, and only 87.0% (n = 1,101) of case patients were overlapping between these two phenotyping algorithms. Even though the PPV of DD ICD-10 code was reported as high as 0.98 [35], we found that considerable phenotyping heterogeneity existed without the supporting procedure reports.

### Genetic associations with DD

**Fig 2:**
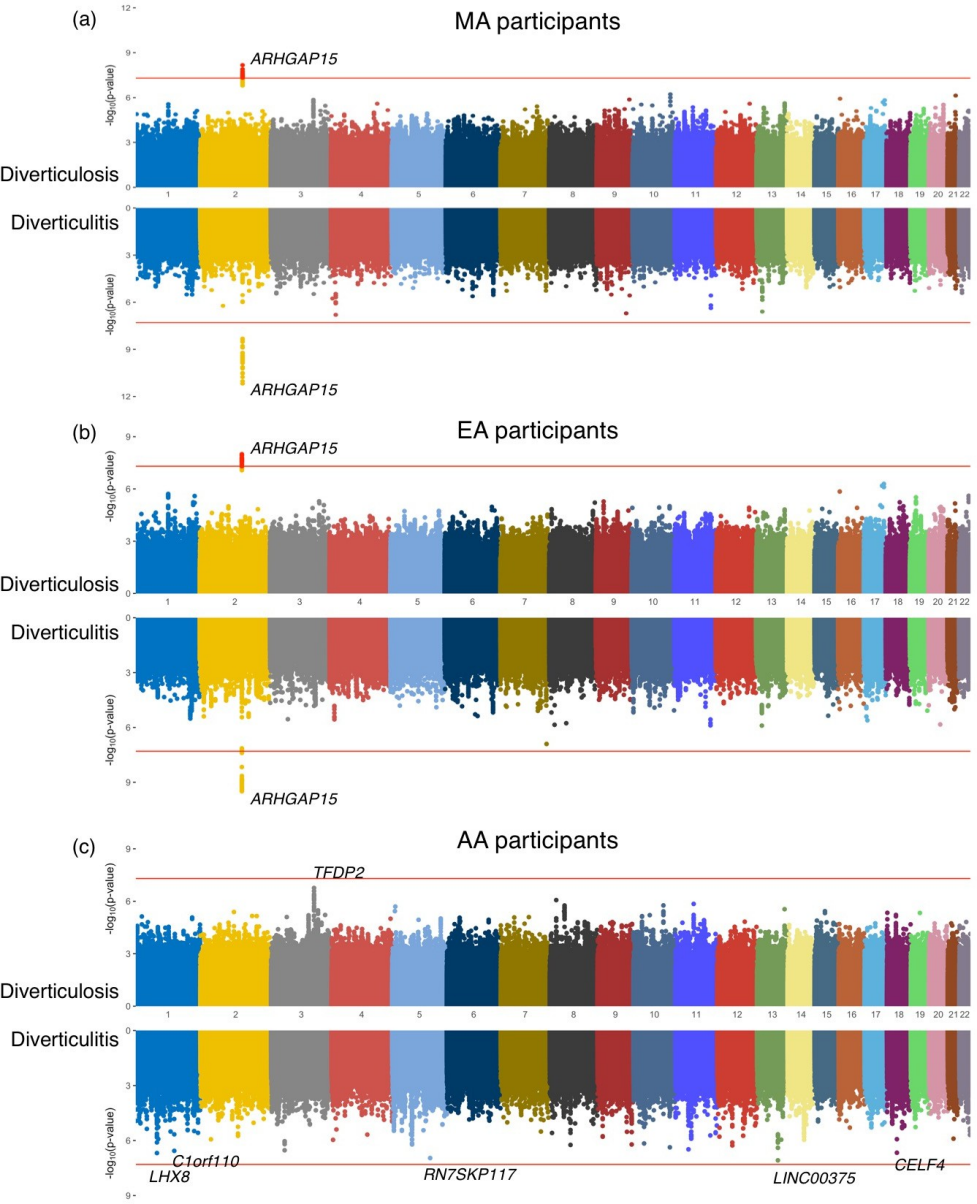
Manhattan plots of genome-wide associations with diverticular disease (DD) in (a) Multiancestry (MA) participants (n = 21,777), (b) European Ancestry (EA) participants (n = 19,211), and (c) African Ancestry (AA) participants (n = 2,322). In each panel, the upper graph presents GWAS results of diverticulosis, and the bottom graph shows GWAS results of diverticulitis. The red horizontal line indicates genome-wide significance of p<5.0E-08 for each analysis.

The GWAS of DD in the MA population identified one genome-wide significant locus **(Fig 2**) at 2q22.3 within *ARHGAP15* gene. The association patterns between the two conditions are largely similar; the diverticulitis GWAS showed more significant and larger ORs than the diverticulosis GWASs in general (**Table 3**). In the MA GWAS for diverticulosis, rs2835676 (*DSCR9* gene) showed strong eQTL association with both transverse and sigmoid colon tissues within the *PIGP* and *TTC3* genes (FDR<3.90E-13).

**Table 3:** Genetic variants that reach suggestive genome-wide significance (P < 1E-06) with diverticulosis or diverticulitis in MA (multi-ancestry), EA (European ancestry) and AA (African ancestry) participants.

	**SNP**	**CHR**	**POS**	**Effect Allele**	**EAF**	**P-value**	**OR**	**SE**	***Nearest Genes***	**Function**	**CADD** *	**RDB** **
Diverticulosis	**rs6736741**	**2**	**144278534**	**C**	**0.180**	**6.77E-09**	**1.17**	**0.03**	***ARHGAP15*:*RP11-570L15*.*2*:*RP11-570L15*.*1***	**ncRNA_intronic**	**0.021**	**5**
MA	rs11200204	10	123633725	A	0.048	6.00E-07	1.26	0.05	*ATE1*	intronic	0.912	6
	rs2835676	21	38591311	T	0.366	7.29E-07	0.90	0.02	*DSCR9*	ncRNA_intronic	0.859	1f
Diverticulitis	**rs10928187**	**2**	**144352544**	**G**	**0.225**	**6.63E-12**	**1.42**	**0.05**	***ARHGAP15*:*RP11-570L15*.*1***	**ncRNA_intronic**	**0.004**	**7**
MA	rs56116508	4	12669861	C	0.104	1.58E-07	1.44	0.07	*RP11-352E6*.*1*	intergenic	3.290	7
	rs9565028	13	35704950	C	0.415	2.53E-07	0.79	0.05	*NBEA*	intronic	0.173	6
	rs77643000	11	118749563	C	0.131	4.23E-07	1.38	0.06	*CXCR5*	intergenic	3.446	5
Diverticulosis	**rs6736741**	**2**	**144278534**	**C**	**0.180**	**1.02E-08**	**1.19**	**0.03**	***ARHGAP15*:*RP11-570L15*.*2*:*RP11-570L15*.*1***	**ncRNA_intronic**	**0.021**	**5**
EA	rs112625468	17	76192318	T	0.443	5.15E-07	0.89	0.02	*AFMID*	intronic	0.272	7
	rs2452920	17	70949903	C	0.377	6.59E-07	0.89	0.02	*SLC39A11*	intronic	3.624	5
Diverticulitis	**rs4662208**	**2**	**144338448**	**A**	**0.177**	**3.22E-10**	**1.46**	**0.06**	***ARHGAP15*:*RP11-570L15*.*1***	**ncRNA_intronic**	**0.922**	**7**
EA	rs12671172	7	152277591	G	0.185	1.25E-07	0.67	0.08	*AC104843*.*4*	intergenic	NA	7
Diverticulosis	rs11569231	3	141692381	C	0.127	1.74E-07	1.76	0.11	*TFDP2*	intronic	2.619	NA
AA	rs114257184	8	12935767	A	0.145	8.56E-07	1.68	0.11	*DLC1*	intergenic	3.604	6
Diverticulitis	rs7327483	13	85722171	C	0.297	8.33E-08	2.55	0.17	*LINC00375*	intergenic	3.128	4
AA	rs140843945	5	124671362	A	0.028	1.11E-07	11.83	0.47	*RN7SKP117*	intergenic	4.134	7
	rs143460556	1	75625398	G	0.033	2.09E-07	5.73	0.34	*LHX8*	intronic	7.885	6
	rs80233487	18	34877201	A	0.023	2.17E-07	6.33	0.36	*CELF4*	intronic	2.813	5
	rs4657237	1	162880767	C	0.415	2.75E-07	2.46	0.18	*C1orf110*	intergenic	2.381	5
	rs6793498	3	39436360	G	0.181	2.96E-07	2.78	0.20	*SLC25A38*	intronic	2.778	7
	rs144422193	11	39890225	G	0.057	3.32E-07	3.93	0.27	*RP11-810F22*.*1*	intergenic	0.209	6
	rs78108838	10	119740292	T	0.070	4.29E-07	3.87	0.27	*RAB11FIP2*	intergenic	0.061	6
	rs74592875	8	58014100	A	0.044	5.79E-07	3.96	0.28	*RNA5SP266*	intergenic	3.428	7
	rs140818624	9	136111965	A	0.014	6.12E-07	10.53	0.47	*LCN1P1*	intergenic	0.563	5
	**rs142519617**	**5**	**64307317**	**G**	**0.012**	**6.19E-07**	**12.24**	**0.50**	***CWC27***	**intronic**	**2.053**	**7**
	rs4749487	10	30034377	G	0.051	6.95E-07	3.11	0.23	*SVIL*	intergenic	11.440	5
	rs11168732	12	49164120	C	0.455	7.81E-07	2.35	0.17	*LINC00935*, *ADCY6*, *MIR4701*	intronic	1.338	5

· Boldface type indicates the variants that meet the genome wide significance. (p-value < 5E-08).

· * deleteriousness score (CADD score 43)

· **potential regulatory functions (RegulomeDB score44)

The genetic signals found from EA-specific analysis and MA analysis were largely analogous, possibly because approximately 85.0% of the discovery population was EA **(Fig 2, Tables 1 and 3)**. Even though *ARHGAP15* loci showed non-significant p-values of 0.24–0.99 in the AA GWAS, the effect directions of *ARHGAP15* loci were mostly positive and substantial, ranging from 1.111 to 1.464 in AA diverticulitis GWAS, except few loci which showed negative ORs **(S3 Table)**.

We performed additional GWASs in the ICD-phenotyped diverticulitis cohort and replicated an *ARHGAP15* locus on chromosome 2 (rs6717024) as genome-wide significant. One of the nearly significant associations included rs11843418 (*FAM115A*), which was previously identified [17, 19] but not significantly detected in our NLP-enriched GWAS possibly due to statistical power or varying genetic composition of study cohorts **(S4 Table)**.

### PheWAS

#### (1) DD susceptibility variants identified in our MA, EA, AA GWAS (p<1E-06) tested in the medical phenome of MA, EA, AA participants

We observed FDR-significant PheWAS associations (FDR < 0.05) between DD phecodes (562, 562.1, and 562.2) and several independent (LD r^2^<0.1) *ARHGAP15* loci in MA and EA PheWAS **(Table 4)**. Other than diverticular EHR phenotypes, rs9565028 (*NBEA* gene) showed FDR-significant associations with genitourinary manifestations including ‘functional disorders of bladder’ and ‘other disorders of bladder’ in the MA and EA phenome. No significant associations were identified in AA PheWAS.

**Table 4:** Significant genotype-EHR phenotype associations (suggestive threshold P<1E-04) from ancestry-stratified PheWAS of the discovered diverticular disease susceptibility SNPs from our GWAS.

	Phecode	SNP	CHR	POS	Effect Allele	Nearest Genes	EHR Phenotype	Category	OR	95% CI	P	Total sample counts	Case counts	Allele Frequency	FDR (<0.05)
**MA participants**	562.1	rs6736741	2	144278534	C	*ARHGAP15*	Diverticulosis	digestive	1.16	(1.12,1.20)	6.93E-17	71835	15883	0.17	TRUE
	562	rs6736741	2	144278534	C	*ARHGAP15*	Diverticulosis and diverticulitis	digestive	1.15	(1.11,1.19)	5.79E-16	72751	16799	0.17	TRUE
	562.2	rs6736741	2	144278534	C	*ARHGAP15*	Diverticulitis	digestive	1.30	(1.21,1.38)	1.11E-14	59080	3128	0.17	TRUE
	562.1	rs10928187	2	144352544	G	*ARHGAP15*	Diverticulosis	digestive	1.13	(1.09,1.16)	8.53E-13	71835	15883	0.21	TRUE
	562	rs10928187	2	144352544	G	*ARHGAP15*	Diverticulosis and diverticulitis	digestive	1.12	(1.08,1.15)	8.99E-12	72751	16799	0.21	TRUE
	562.2	rs10928187	2	144352544	G	*ARHGAP15*	Diverticulitis	digestive	1.24	(1.16,1.32)	9.26E-12	59080	3128	0.21	TRUE
	596.5	rs9565028	13	35704950	C	*NBEA*	Functional disorders of bladder	genitourinary	0.88	(0.83,0.93)	3.48E-06	80967	2958	0.37	TRUE
	596	rs9565028	13	35704950	C	*NBEA*	Other disorders of bladder	genitourinary	0.91	(0.88,0.95)	8.73E-06	83963	5954	0.37	TRUE
	182	rs9565028	13	35704950	C	*NBEA*	Malignant neoplasm of uterus	neoplasms	0.82	(0.74,0.90)	4.89E-05	77013	952	0.37	FALSE
	365.1	rs77643000	11	118749563	C	*CXCR5*	Open-angle glaucoma	sense organs	1.16	(1.08,1.25)	8.86E-05	76485	3529	0.12	FALSE
**EA participants**	562.1	rs386651361	2	144338448	A	*ARHGAP15*	Diverticulosis	digestive	1.18	(1.13,1.22)	2.96E-16	53892	13716	0.17	TRUE
	562.1	rs6736741	2	144278534	C	*ARHGAP15*	Diverticulosis	digestive	1.17	(1.13,1.22)	5.43E-16	53892	13716	0.18	TRUE
	562	rs386651361	2	144338448	A	*ARHGAP15*	Diverticulosis and diverticulitis	digestive	1.17	(1.12,1.21)	2.09E-15	54524	14348	0.17	TRUE
	562	rs6736741	2	144278534	C	*ARHGAP15*	Diverticulosis and diverticulitis	digestive	1.16	(1.12,1.21)	1.01E-14	54524	14348	0.18	TRUE
	562.2	rs386651361	2	144338448	A	*ARHGAP15*	Diverticulitis	digestive	1.32	(1.23,1.42)	1.45E-14	42850	2674	0.17	TRUE
	562.2	rs6736741	2	144278534	C	*ARHGAP15*	Diverticulitis	digestive	1.31	(1.22,1.40)	1.40E-13	42850	2674	0.17	TRUE
	562.2	rs13409480	2	144403796	A	*ARHGAP15*	Diverticulitis	digestive	0.85	(0.80,0.90)	9.60E-09	42850	2674	0.50	TRUE
	526.4	rs191450774	1	210789175	T	*HHAT*	Temporomandibular joint disorders	digestive	1.69	(1.32,2.15)	2.60E-05	59771	1927	0.01	FALSE
	562	rs13409480	2	144403796	A	*ARHGAP15*	Diverticulosis and diverticulitis	digestive	0.94	(0.92,0.97)	6.39E-05	54524	14348	0.50	FALSE
**AA participants**	253.3	rs80233487	18	34877201	A	*CELF4*	Diabetes insipidus	endocrine/metabolic	6.03	(2.79,13.01)	4.68E-06	11484	35	0.02	FALSE
	287.1	rs4657237	1	162880767	T	*-*	Spontaneous ecchymoses	hematopoietic	0.48	(0.34,0.67)	2.27E-05	11501	76	0.53	FALSE
	743.13	rs6793740	3	141772040	A	*TFDP2*	Other specified osteoporosis	musculoskeletal	1.46	(1.22,1.74)	3.47E-05	11840	292	0.26	FALSE
	333	rs11168732	12	49164120	C	*LINC00935*, *ADCY6*, *MIR4701*	Extrapyramidal disease and abnormal movement disorders	neurological	1.42	(1.20,1.68)	4.45E-05	11046	290	0.40	FALSE
	840.2	rs4749487	10	30034377	G	*-*	Rotator cuff (capsule) sprain	injuries & poisonings	1.71	(1.31,2.25)	9.86E-05	11039	265	0.08	FALSE

*AF = Allele Frequency

#### (2) DD susceptibility variants identified in previous GWAS (p < 5E-08) tested in the medical phenome of MA, EA, AA participants

In the MA PheWAS, 55 genotype-EHR phenotype associations were significant **(S5 Table)**. Among them, 18 significant genotype-EHR associations were endocrine/metabolic phenotypes, 17 of them were digestive phenotypes and 10 of them were circulatory system related phenotypes. The largest number of significant EHR phenotype associations were DD; 7 ‘diverticulosis and diverticulitis’, 7 ‘diverticulosis’ and 1 ‘diverticulitis’ were identified as significant. Other than the *ARHGAP15* loci, rs4333882 (*SLC35F3* gene) and rs10472291 (*WDR70* gene) showed significant clinical associations with DD. SNP rs9272785 (*HLA-DQA1* gene, proxy variant for rs7990) generated the most significant association in MA PheWAS coupled with ‘rheumatoid arthritis’. The SNP was also strongly associated with several diabetes manifestations, including ‘type 1 diabetes’, ‘type 1 diabetes with ophthalmic’, ‘type 1 diabetes with ketoacidosis’, ‘type 2 diabetes’, etc.

In the EA PheWAS, 49 genotype-EHR phenotype associations were identified with FDR significance **(S5 Table)**. Among them, 17 EHR phenotypes are classified as digestive phenotypes, 15 are endocrine/metabolic-related phenotypes and 6 were related to the circulatory system. Rs9272785 (*HLA-DQA1* gene) also marked the most significant association in EA PheWAS with ‘rheumatoid arthritis’. The variant also revealed additional associations in the EA phenome, including ‘developmental delays and disorders’, ‘multiple sclerosis’, ‘ulcerative colitis’ and ‘chronic lymphocytic thyroiditis’.

In AA PheWAS, two genotype-EHR phenotype associations met FDR significance: rs9272785 (*HLA-DQA1* gene) showed the most significant SNP-phenotype association as it did in MA and EA PheWAS. The variant also showed strong associations with ‘type 1 diabetes with ketoacidosis’ and ‘type 1 diabetes’ in the AA phenome.

## Discussion

To date, patient identification in the EHR was partially limited in that mostly inpatient medical coding was used, which might result in under-diagnosis of the case patients and/or misclassification of controls who possibly have DD. In the most recent GWAS of DD [18], the review of replication cohorts and input of physicians/technicians were manual; however, manual review has limited application to larger population-based datasets in its lack of scalability. Our NLP-enriched phenotyping approach showed a significant improvement in performance (algorithm PPVs≥0.94, 3.5-fold increase in diverticulosis patient identification) compared with the use of only ICD-codes, **(Table 2)** and supports the importance of leveraging the full breadth of data captured in the her [36, 37].

Our multi-ancestry GWAS of DD confirmed the strong genome-wide association of *ARHGAP15* with both diverticulosis and diverticulitis **(Table 3)**. *ARHGAP15* is known to strongly and negatively regulate GTPase binding property of the Rac protein family in leukocytes, which modulates important antimicrobial functions [38]. This mechanism of *ARHGAP15* possibly impacts the inflammatory environment of the intestine, promoting the development of diverticula or progression of diverticula due to bacterial growth along the colonic wall. In the ancestry-stratified GWAS analyses, the often-replicated associations between *ARHGAP15* with DD was detected in EA cohorts and similarly positive effect sizes but little to no association was observed in AA cohorts **(S3 Table)**. Notably, the sample size for the AA cohort is less than 1/10^th^ that of the EA cohort, as well as different risk allele frequencies between ancestries. Our additional power calculation showed that at least 15,000 participants are needed to perform GWAS on the *ARHGAP15* loci (EAF 0.18, disease prevalence 0.10, OR 1.20) with 80% statistical power **(S1 Fig)**. Further investigation is needed to confirm the universal susceptibility effect of *ARHGAP15* to DD in patient of non-European ancestry.

Our PheWAS of the independent *ARHGAP15* loci (rs6736741, rs10928187, rs386651361) confirmed its significant phenotypic expression with DD in MA and EA and the second most significant association with paralytic ileus **(Table 4)**. Some genitourinary phenotypes of functional bladder disorders found in MA and EA should be noted in that the muscular motility or neuromuscular dysfunction of internal organs possibly influence both colonic walls for diverticulosis and bladder muscle for urinary disorders.

In the PheWAS of the established diverticular variants, we identified several circulatory system related EHR phenotypes associated with DD variants, including phlebitis and thrombophlebitis, pulmonary heart disease, and deep vein thrombosis. Notably, recent studies have suggested a possible epidemiologic association between DD and acute coronary syndromes and thromboembolic events [39, 40]. We also confirmed the associations of rs9272785 (*HLA-DQA1* gene) with type 1 diabetes and its manifestations with FDR significance across ancestries. The HLA class 2 region, where rs9272785 is located, is not only associated with risk of type 1 diabetes but also increased susceptibility to juvenile rheumatoid arthritis and other autoimmune diseases [41, 42].

**Fig 3:**
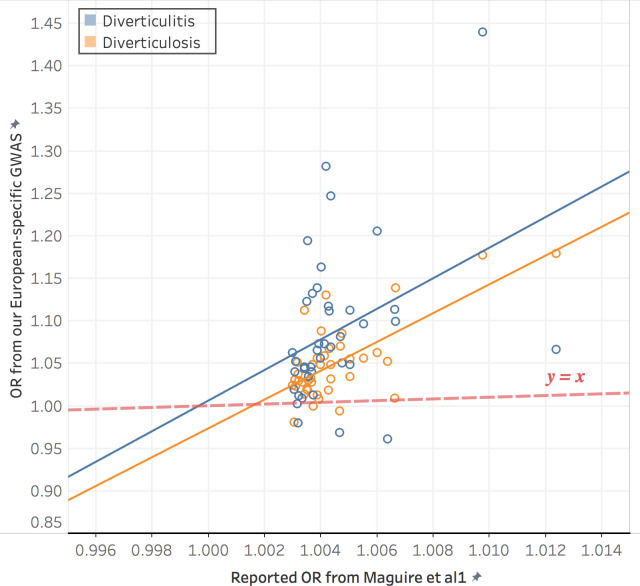
Comparison of effect size (OR) between our GWAS with NLP-enriched phenotyping and previous GWAS with ICD-based phenotyping from Maguire et al. The dashed y = x line indicates equal ORs in both studies.

Compared to previous GWASs of DD, our summary statistics generally show larger effect sizes possibly fueled with the improved patient identification by the NLP-enriched phenotyping algorithm. For example, rs6734367, the strongest *ARHGAP15* locus reported in Maguire et al. [17] showed positive OR of 1.010 in the original study, whereas it presents an OR as high as 1.177 (diverticulosis) and 1.280 (diverticulitis) in our EA GWAS with the same allelic direction **(S6 Table)**. For the rest of the GWAS-significant SNPs, the ORs in our results generally showed increased effect sizes despite a cohort 1/20^th^ the size of Maguire et al. **(Fig 3)**. Among the 52 tested variants, 5 loci were significantly replicated in our EA GWAS of diverticulosis (p-value < 0.05/52). As the cohort size gets larger, and patients with diverse genetic backgrounds are included, our results suggest improved analytical power for future genomic research with the integration of different layers of EHR data.

There are several caveats in our study. We did not separately validate our phenotyping algorithms’ performance for diverticulitis vs. diverticulosis, which should be included in future research. Our GWAS did not identify any novel association and only confirmed an existing locus with DD, albeit with larger effect sizes across the analyses. Also, our MA analysis was composed of 85% EA participants, so the signals are largely driven by EA-centric results. The cohort size of AA is considerably smaller than the EA or MA cohorts, which elevates the risk for false positive findings.

Our approach has highlighted the richness and potential of the heterogenous EHR data in patient classification with NLP, and the feasibility of an integrative analytical pipeline, from GWAS to post-GWAS analysis such as PheWAS, to facilitate etiological investigation of a disease in clinical setting.

## Supporting information

S1 Fig Results of power calculation for our DD GWAS analyses. (TIF)

S2 Fig QQ plots of our DD GWAS results. (TIF)

S1 Table PheWAS association results (p-value < 1E-04) of 52 susceptibility SNPs for diverticular diseases in MA, EA, AA participants. (XLSX)

S2 Table List of exclusion ICD codes for phecode mapping: Not classified as control or case. (XLSX)

S3 Table GWAS results of ARGHAP loci among participants of African ancestry. (XLSX)

S4 Table GWAS results of ICD-based identified patients with diverticulitis in eMERGE cohort. (XLSX)

S5 Table Information of 52 reported susceptibility variants from three Eurocentric GWAS of diverticular diseases: Sid et al. (2017), Maguire et al. (2018), and Sch et al. (2019).
(XLSX)

S6 Table OR comparison between our European-specific GWASs and the previous GWAS results from Maguire et al. (XLSX)

S1 File Details of genotyping, imputation, and quality control processes. (DOCX)
