# Supplementary figures and images for: Multi-ancestry genome- and phenome-wide association studies of diverticular disease in electronic health records with natural language processing enriched phenotyping algorithm
Source: PLoS One. 2023 May 17;18(5):e0283553. doi: 10.1371/journal.pone.0283553 (PMC10191288; doi:10.1371/journal.pone.0283553)

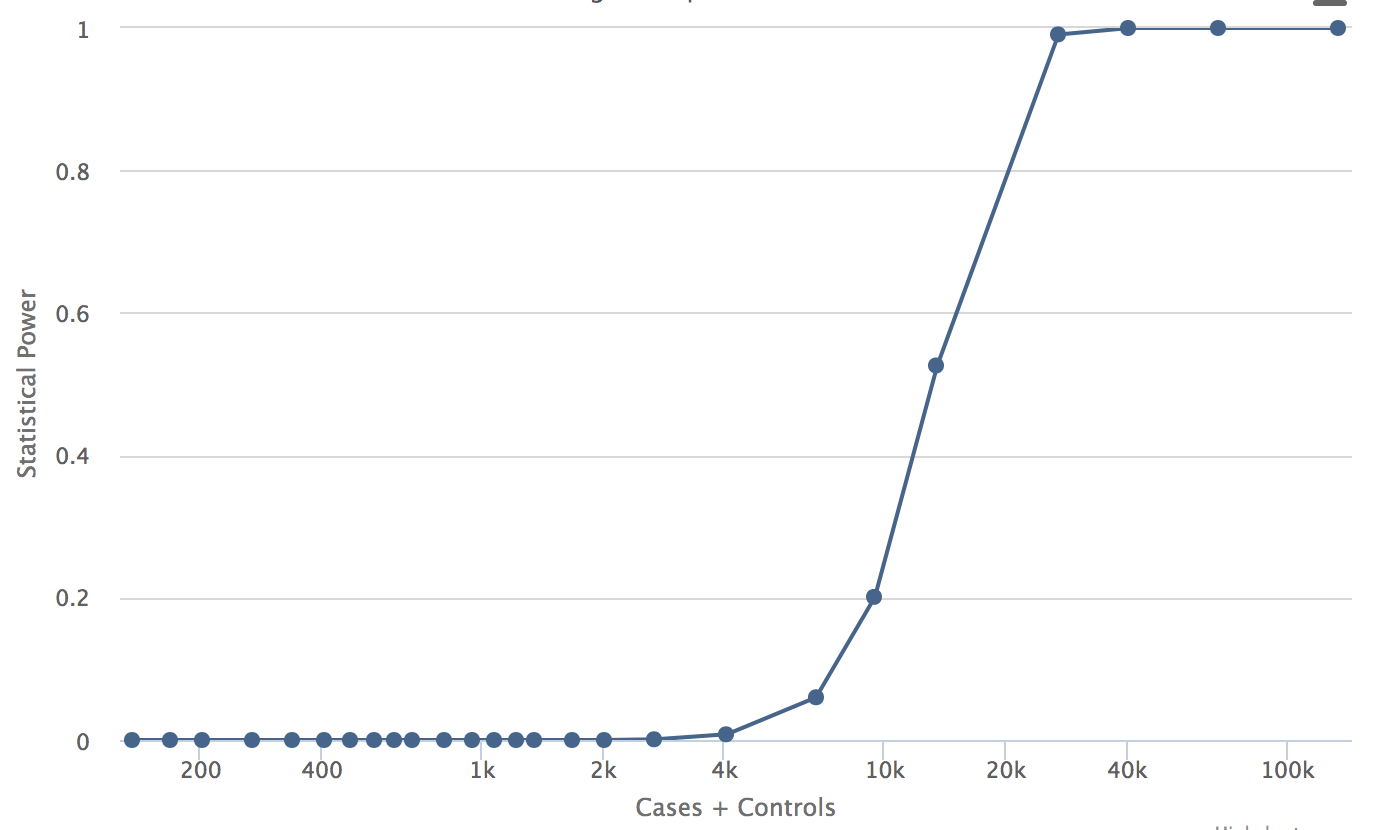

Supplement: S1 Fig — (TIF) [file pone.0283553.s001.tif]

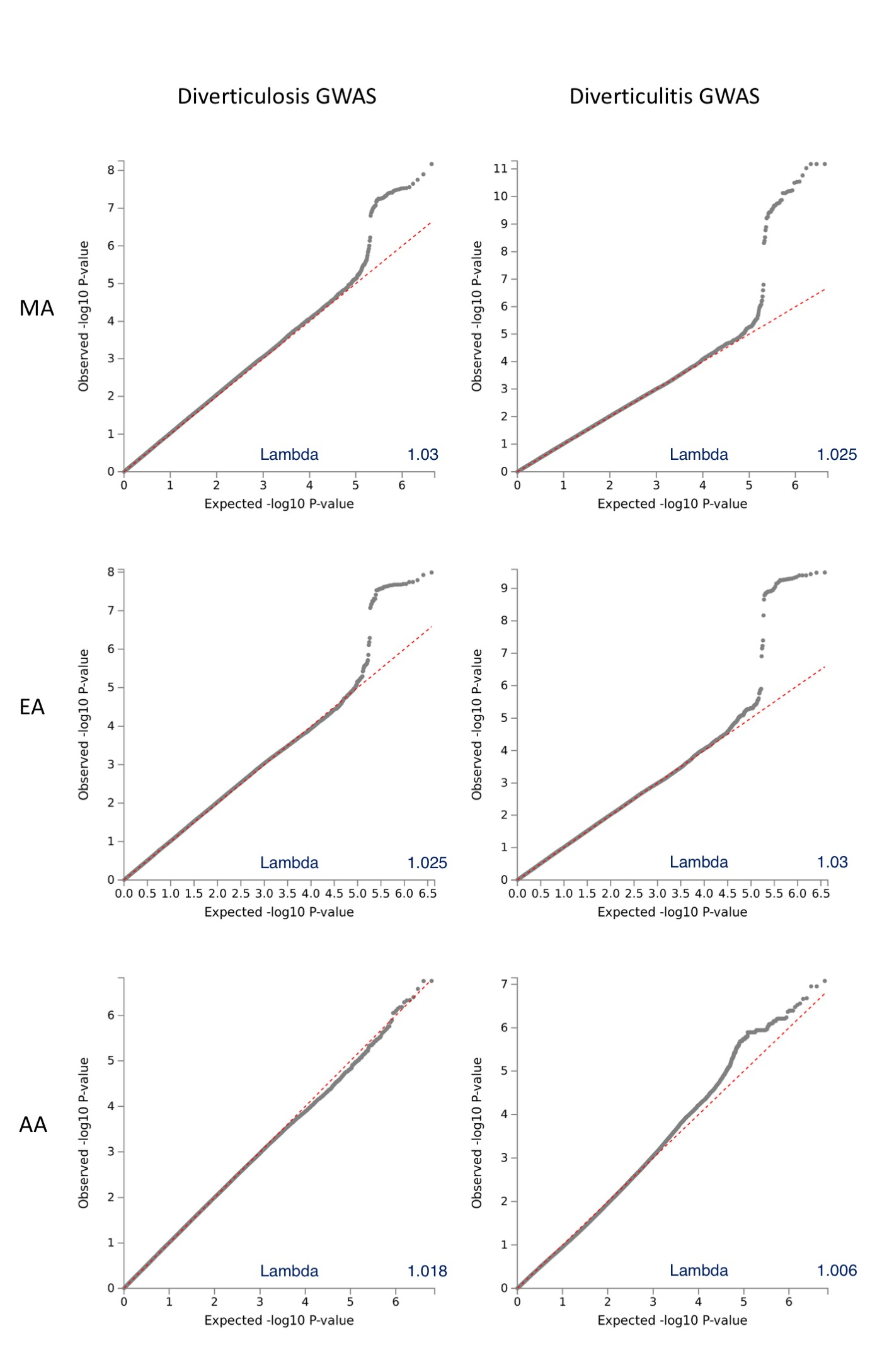

Supplement: S2 Fig — (TIF) [file pone.0283553.s002.tif]
