# Supplementary material for: Multi-ancestry genome- and phenome-wide association studies of diverticular disease in electronic health records with natural language processing enriched phenotyping algorithm
Source: PLoS One. 2023 May 17;18(5):e0283553. doi: 10.1371/journal.pone.0283553 (PMC10191288; doi:10.1371/journal.pone.0283553)
Supplement: S1 File — (DOCX) [file pone.0283553.s009.docx]

***S1 Method.***

***Study population***

Genome-wide genotype data of 38,827 individuals from 9 biobanks and phenotype data including their demographic, clinical diagnosis, colonoscopy reports of 99,185 individuals were collected from 12 biobanks in the eMERGE consortium^1^. The genetic ancestry of each participant was determined by the intersection of self-reported race and principal component analysis (PCA) based k-mean clustering. After identity by descent (IBD) analysis, we removed 8,019 related individuals that were not in canonical IBD position or genetically identical individuals near the origins (Z0 > 0.83 and Z1 < 0.1). We also obtained basic demographic and other patient information for covariate adjustment: sex, age and body mass index (BMI) at first diagnosis of diverticular disease for cases where available; or if not available, BMI at time of last colonoscopy which was also used for controls; or median BMI if none of the above BMI measures were available.

***Genotyping data and QC process***

The DNA samples of the participants were genotyped on 78 Illumina or Affymetrix array batches collected from 12 medical sites. Genotypes were imputed using the Haplotype Reference Consortium (HRC1.1)^2^, which resulted in ~40 million genetic variants: details of this imputation including QC were published separately by the eMERGE network^1^. We filtered the poorly imputed genetic variants with the r-squared imputation quality threshold (mean variant R^2^) < 0.3, minor allele frequency (MAF) < 0.01 and genotype call rate < 90%, which resulted in 9,848,164 autosomal polymorphic variants for analysis.

***NLP-enriched phenotyping algorithm for diverticular disease***

For patients with colonoscopy or abdominal imaging reports in the EHR, we developed and validated an NLP algorithm **(Figure 1a)** to identify diverticular disease and implemented this algorithm across five eMERGE sites (Northwestern University (NU), Vanderbilt University (VU), Geisinger, Kaiser Permanente Washingon / University of Washington (KPWA/UW), and Mayo Clinic). The NLP algorithm considered any subject that had any positively asserted mention of “diverticul*” in those reports to have diverticulosis, and a positively asserted mention of “diverticulitis” was considered to have diverticulosis with diverticulitis. We used the NegEx or similar algorithm^3^ to detect negated mentions of either diverticulosis or diverticulitis. For example, if a patient had a note which contained a variation of the phrase “diverticulosis without evidence of diverticulitis,” the patient was considered to have diverticulosis but not diverticulitis; or, if a patient had a procedure note which contained the phrase “no diverticula found,” then the patient was considered to be a control without diverticulosis or diverticulitis. In addition, any patient who had a colonoscopy or abdominal imaging, and within 7 days after the procedure had International Classification of Disease 9^th^ revision (ICD-9) codes 562.01 ‘Diverticulitis of small intestine’, 562.03 ‘Diverticulitis of small intestine with hemorrhage’, 562.11 ‘Diverticulitis of colon (without mention of hemorrhage)’, or 562.13 ‘Diverticulitis of colon with hemorrhage’) in an inpatient or outpatient setting, were considered to have diverticulitis **(Fig 1a)**. ICD-10 diagnosis codes were not collected as our algorithms were developed and validated before 2015, and final phenotype data was collected soon after ICD-10 CM was implemented in the United States. Finally, we identified controls without diverticulosis as having at least one colonoscopy and no positively asserted mention of “diverticul*” in any colonoscopy or abdominal imaging procedure report.

For two sites (Marshfield, Mount Sinai) where only a limited subset of these imaging reports were available, we instead selected diverticulosis cases, either with or without diverticulitis, as patients with ICD-9 diagnosis codes that started with 562 (‘Diverticulosis and diverticulitis’ category’), assigned within 7 days after a colonoscopy or abdominal imaging **(Fig 1b)**. Diverticulosis with diverticulitis cases had any of the distinct 562 sub-codes for diverticulitis. We defined controls as having no diagnosis codes for diverticulosis or diverticulitis, yet had at least one colonoscopy, and no mention of “diverticul*” in any of the colonoscopy or abdominal imaging procedure reports, when available **(Fig 1b).**

Four sites (NU, VU, Marshfield, Geisinger) validated algorithm performance by a standardized chart review of randomly selected patients’ charts. Trained clinicians and chart reviewers reviewed a total of 225 of diverticulosis cases, both with and without diverticulitis, and 139 controls without diverticulosis (nor diverticulitis), to assess the positive predictive value (PPV) of the case and control algorithms, using chart review and validation procedures established by the eMERGE network^4^. Specifically, to validate our phenotyping algorithms, sites selected a random subset of the charts of patients whom the algorithms determined were cases and controls, and then conducted blinded reviews of those charts either by: 1) having clinicians manually review their charts, to determine if truly diverticulosis cases (with or without diverticulitis) or not; or 2) using software developed by one of the authors (LR) to annotate all positive and negative mentions of “diverticul*” in the randomly selected patients’ colonoscopy and/or abdominal imaging reports, from which case (with or without diverticulitis) or control status was confirmed.

**References**

1. Stanaway IB, Hall TO, Rosenthal EA, et al. The eMERGE genotype set of 83,717 subjects imputed to ~40 million variants genome wide and association with the herpes zoster medical record phenotype. Genet Epidemiol 2019;43:63-81.

2. McCarthy S, Das S, Kretzschmar W, et al. A reference panel of 64,976 haplotypes for genotype imputation. Nat Genet 2016;48:1279-83.

3. Harkema H, Dowling JN, Thornblade T, et al. ConText: an algorithm for determining negation, experiencer, and temporal status from clinical reports. J Biomed Inform 2009;42:839-51.

4. Newton KM, Peissig PL, Kho AN, et al. Validation of electronic medical record-based phenotyping algorithms: results and lessons learned from the eMERGE network. J Am Med Inform Assoc 2013;20:e147-54.
